# Sex-specific asymmetry in lumbar paraspinal muscles among chronic low back pain patients: correlation with pain duration and intensity

**DOI:** 10.1038/s41598-025-89819-y

**Published:** 2025-02-18

**Authors:** Sihai Liu, Luis Becker, Bernhard Hoehl, Sandra Reitmaier, Lukas Mödl, Daishui Yang, Tianwei Zhang, Matthias Pumberger, Hendrik Schmidt

**Affiliations:** 1 Berlin Institute of Health, Julius Wolff Institute for Biomechanics and Musculoskeletal Regeneration, Charité – Universitätsmedizin Berlin, Augustenburger Platz 1, 13353 Berlin, Germany; 2 Center for Musculoskeletal Surgery, Charité – Universitätsmedizin Berlin, Charitéplatz 1, 10117 Berlin, Germany; 3 Institute of Biometry and Clinical Epidemiology, Charité-Universitätsmedizin Berlin, Charitéplatz 1, 10117 Berlin, Germany; 4 Department of Orthopedics, Wuhan Fourth Hospital, 430033 Wuhan, China

**Keywords:** Chronic low back pain, Sex specific, Paraspinal muscles, Asymmetry index, Magnetic resonance imaging, Lumbar spine, Chronic pain, Skeletal muscle

## Abstract

Chronic low back pain (cLBP) is a multifactorial condition, including paraspinal muscle asymmetry. Understanding the relationship between muscle asymmetry and cLBP and how this varies by sex is crucial for targeted interventions. From January 2022 to December 2023, 250 participants were enrolled: 117 without back pain (no-BP) (61 females, 56 males) and 133 with cLBP (69 females, 64 males). MRI assessed the cross-sectional area (CSA), functional CSA (FCSA), and fat infiltration (FI) of the paraspinal muscles, including the psoas major (PM), quadratus lumborum (QL), erector spinae (ES), and multifidus (MF), at all lumbar levels. Asymmetry indices were calculated for CSA (CAI), FCSA (FCAI), and FI (FIAI). Data were analyzed using multiple linear and logistic regression. Muscle asymmetries were observed in both sexes and groups, with significant differences in the CSA and FCSA of the QL in males and notable FI asymmetries in the cLBP group. In women with cLBP, higher ES CAI and FCAI at L5/S1 (p < 0.05) and lower PM FCAI at L4/5 (OR 0.869, 95% CI 1.008–1.150, p = 0.03) were observed. FIAI was significantly higher in cLBP for ES at L2/3 and PM, MF, and ES at L4/5 (p < 0.05). Men with cLBP had lower PM CAI and FCAI at L1/2 and L4/5 (p < 0.05), with varying FIAI levels. FIAI correlated with cLBP duration and intensity in both sexes (p < 0.05). Paraspinal muscle asymmetry, particularly in fat infiltration, is associated with cLBP and varies by sex. These findings support sex-specific approaches to managing cLBP.

## Introduction

Chronic low back pain (cLBP) is a leading cause of functional impairment and disability globally, significantly impacting both, individuals and socioeconomic aspects^1–4^. Despite extensive research into the etiology and management, the pathogenic mechanisms of cLBP remain complex and multifactorial. Among these mechanisms, the relationship between paraspinal muscles and cLBP has garnered increasing attention, with substantial evidence linking the composition and morphological changes of lumbar paraspinal muscles to cLBP^5–8^. However, less focus has been given to the structural and functional changes in muscle, particularly regarding their symmetry.

Previous studies have suggested that asymmetry of paraspinal muscles may contribute to the pathogenesis of cLBP. For instance, Hides et al.^9^ reported significant muscle asymmetry and dysfunction in the multifidus muscles of cLBP patients. Furthermore, systematic reviews indicate that the multifidus and paraspinal muscle groups are considerably smaller in cLBP patients compared to healthy individuals, and the symptomatic multifidus and paraspinal muscles are significantly smaller on the affected side in patients with chronic unilateral cLBP^10^. Conversely, some studies have observed asymmetry in the multifidus (MF) and erector spinae (ES) muscles even in healthy adults^11,12^.

Although existing research has explored the relationship between paraspinal muscle asymmetry and cLBP, findings have been inconsistent and often focused on specific muscles or single parameters. This study aimed to systematically evaluate the relationship between asymmetry indices of various paraspinal muscles—including the psoas major (PM), quadratus lumborum (QL), MF, and ES—and cLBP. To account for these differences, this study will analyze male and female subjects separately. This approach will enable a more accurate assessment of sex-specific impacts on paraspinal muscle asymmetry and facilitate the development of more targeted clinical intervention strategies.

It is important to note that the composition and morphology of paraspinal muscles differ significantly between males and females^13,14^. To account for these differences, this study will analyse males and females separately. This approach will enable a more accurate assessment of sex-specific impacts on paraspinal muscles asymmetry and facilitate the development of more targeted clinical intervention strategies.

We hypothesized that patients with cLBP will exhibit higher asymmetry indices of paraspinal muscles compared to individuals without back pain (no-BP), and this asymmetry will be associated with the duration and intensity of cLBP. Furthermore, we hypothesized that this asymmetry will differ between males and females, necessitating separate evaluation and analysis. A comprehensive understanding of the relationship between lumbar paraspinal muscle asymmetry and cLBP could provide new perspectives for clinical diagnosis and intervention.

## Patients and methods

A large, ongoing population-based observational study collected lumbar MRI scans from 117 individuals with no history of back pain (56 males and 61 females, aged 19 to 64) and 133 patients with cLBP (64 males and 69 females, aged 20 and 63). Demographic data for both groups are presented in Table 1. The study received approval from the local ethics committee (Approval number: EA1/058/21), and all participants provided informed written consent for the use of their data in this research. This study follows the Strengthening the Reporting of Observational Studies in Epidemiology (STROBE) guidelines. The no-BP group consist of individuals who have never experienced pain in the back or pelvis and have not undergone surgery on the spine, pelvis, or hip joints. The cLBP group includes patients with persistent nonspecific LBP lasting more than 12 weeks. Exclusion criteria for the study were body mass index greater than 28 kg/m^2^, prior vertebral fractures, radiculopathies causing muscle weakness, previous spinal surgery, or non-spinal conditions that significantly reduce daily activity (such as cardiovascular diseases, heart failure, myocardial ischemia, neurological disorders, or malignancies) were not included in this study.

**Table 1:** Participant demographics (numbers are given as mean $\pm$ SD).

	no-BP		cLBP	
Sex	Males	Females	Males	Females
Age (Years)	41.52 $\pm$ 13.54	40.34 $\pm$ 11.83	41.17 $\pm$ 10.45	42.54 $\pm$ 10.81
BMI (kg/m^2^)	23.83 $\pm$ 2.24	22.35 $\pm$ 2.04	24.06 $\pm$ 2.18	23.11 $\pm$ 2.36
Pain Intensity	–	–	3.86 $\pm$ 1.56	4.25 $\pm$ 1.33
Pain Duration (Years)	–	–	6.84 $\pm$ 6.81	12.43 $\pm$ 8.67

An orthopedic surgeon gathered information about the participants’ age, sex, height, and weight through interviews and physical measurements. The intensity of cLBP was assessed using a 11-point numeric rating scale (NRS: 0 = “no pain” to 10 = “worst pain”), and the duration of cLBP was also recorded. All patients recorded a minimum pain duration of three months, and the pain intensity was determined based on the average pain intensity over the past three months.

Optimal differentiation among the various muscles in the lumbar spine was achieved using a Siemens Avanto 3.0 T MRI system (Siemens AG, Erlangen, Germany). Axial and sagittal images were acquired with T2-weighted turbo spin echo sequences. For the axial T2 images, the parameters included a repetition time of 4.000, an echo time of 113 ms, and a slice thickness of 3 mm. All measurements were conducted twice by an orthopedic resident who received specialized training in MRI muscle assessment. Image J (version 1.53, National Institutes of Health, Bethesda, Maryland, USA) was employed for measuring and segmenting MRI images using an established automatic thresholding technique^15^.

**Fig. 1:**
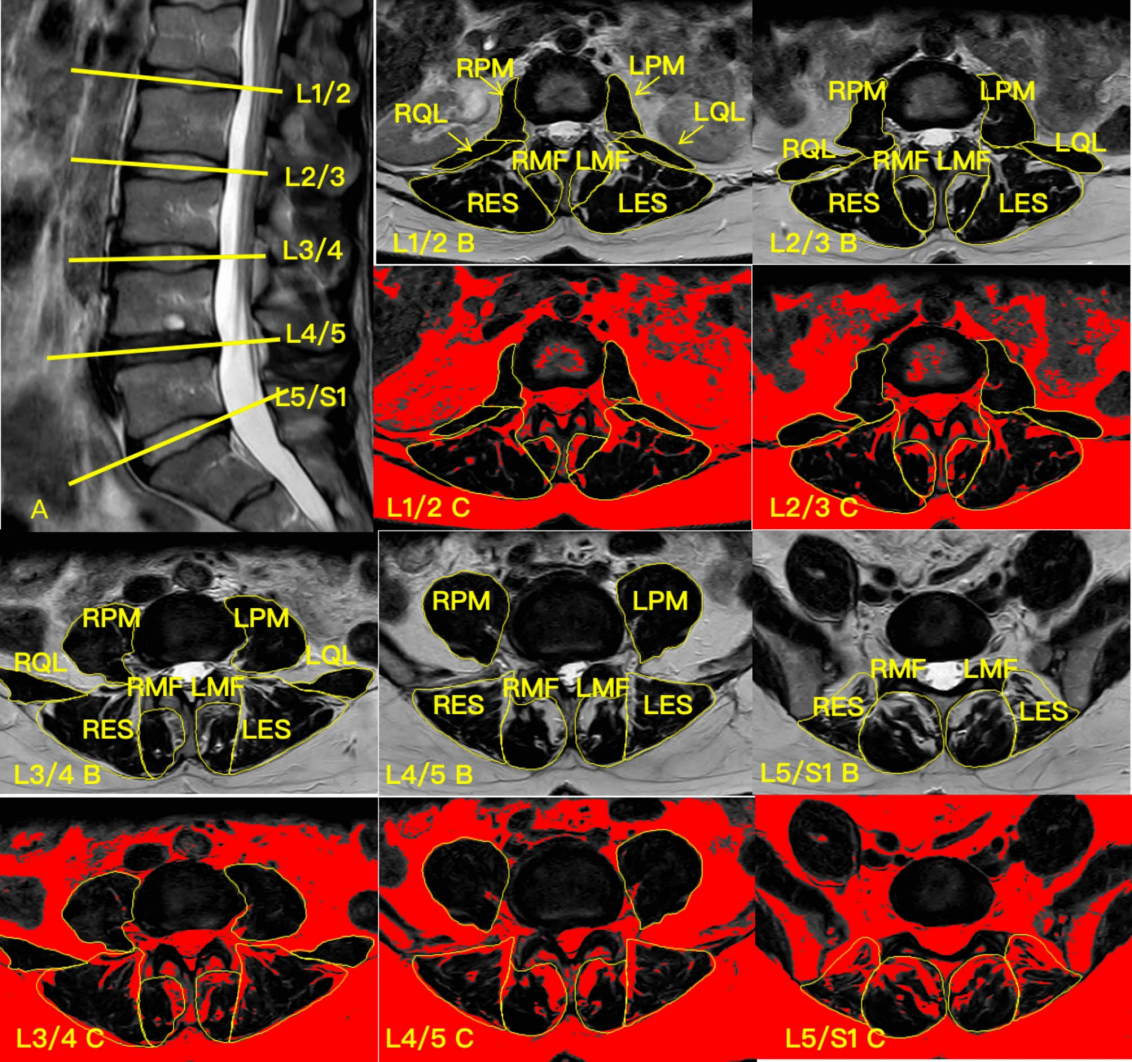
MRI of a subject’s lumbar spine and target muscle measurements. (**A**) Sagittal view of the lumbar spine, with the yellow line indicating the location of the measured cross-sectional MRI; (**B**) Cross-sectional area of the psoas major (PM), quadratus lumborum (QL), multifidus (MF), and erector spinae (ES) at the L1/2 segment; (**C**) Functional cross-sectional area of the PM, QL, MF and ES at the L1/2 segment. *LPM* left psoas major, *LQL* left quadratus lumborum, *LMF* left multifidus, *LES* left erector spinae, *RPM* right psoas major, *RQL* right quadratus lumborum, *RMF* right multifidus, *RES* right erector spinae.

The cross-sectional area (CSA) and functional cross-sectional area (FCSA) of the PM, QL, MF, and ES were assessed at the mid-disc level of all lumbar spinal segments (Fig. 1). Due to measurement limitations, the PM muscles were evaluated from L1/2 to L5/S1 and the QL from L1/2 to L3/4. FI was calculated using the formula FI = ((CSA − FCSA)/CSA)*100. Three key indices were used to assess paraspinal muscle asymmetry: the CSA asymmetry Index (CAI), FCSA asymmetry Index (FCAI), and FI asymmetry Index (FIAI). These indices quantify the asymmetry between the left and right sides of the muscles:

$$\text{CSA Asymmetry Index }(\text{CAI}) = \left| \left(\text{Left CSA}-\text{Right CSA}\right)/\left(\text{Left CSA}+\text{Right CSA}\right)\right|\times 100$$

$$\text{FCSA Asymmetry Index }(\text{FCAI}) = \left|\text{Left FCSA}-\text{Right FCSA}/\text{Left CSA}+\text{Right CSA}\right|\times 100$$

$$\text{FI Asymmetry Index }(\text{FIAI}) = \left|\text{ Left FI}-\text{Right FI}\right|$$

Descriptive statistics were used to compute baseline demographic and clinical variables. Intra-rater reliability was assessed using intraclass correlation coefficients, all exceeding 0.75, indicating excellent consistency according to Portney and Watkins^16^. Paraspinal muscle parameters (CSA, FCSA, and FI) were compared between the left and right sides using multiple linear regression. Binary logistic regression was applied to examine differences in asymmetry indices between the no-BP and cLBP groups. Multiple linear regression was also used to investigate the relationships between asymmetry indices and cLBP duration and intensity. All multivariate models were adjusted for age and BMI. Effect sizes (eta squared). were categorized based on Cohen’s^17^ conventions: small (0.01), medium (0.06), and large (0.14). The statistical analyses were conducted using SPSS version 23.0 (SPSS Inc, Chicago, Illinois).

## Results

### Differences between left and right paraspinal muscles in the no-BP group

Table 2 shows that in the no-BP group, both males and females exhibit asymmetry in CSA, FCSA, and FI between the left and right sides. However, males display more significant asymmetry compared to females. In females, significant asymmetry was observed only in the CSA of the QL at L3/4 (P = 0.047, Partial η^2^ = 0.03) (Supplementary Table 1). In contrast, males exhibited statistically significant differences in the CSA and FCSA of the QL at L2/3 (P = 0.02, Partial η^2^ = 0.05) and L3/4 (P = 0.04, Partial η^2^ = 0.04). No other parameters showed significant differences.

**Table 2:** Differences between left and right paraspinal muscles in the no-BP group.

	CSA, mean (SD)		p value	FCSA, mean (SD)		p value	FI (%), mean (SD)			p value
	Left	Right		Left	Right		Left	Right		
Females										
LI/2 level										
PM	2.51 (1.06)	2.33 (1.00)	0.36	2.46 (1.04)	2.29 (0.98)	0.36	1.85 (3.13)	1.85 (2.94)		1.00
QL	1.60 (0.65)	1.52 (0.62)	0.48	1.57 (0.64)	1.49 (0.61)	0.47	1.85 (3.13)	1.94 (2.44)		0.86
MF	2.20 (0.54)	2.26 (0.52)	0.54	1.99 (0.49)	2.06 (0.49)	0.40	8.93 (9.64)	8.51 (7.40)		0.77
ES	15.6 (2.84)	15.4 (2.90)	0.65	14.8 (2.64)	14.6 (2.69)	0.66	5.32 (3.57)	5.23 (3.22)		0.88
L2/3 level										
PM	5.79 (1.36)	5.77 (1.43)	0.94	5.71 (1.36)	5.68 (1.43)	0.90	1.41 (2.33)	1.67 (2.63)		0.55
QL	2.44 (0.90)	2.25 (0.79)	0.19	2.38 (0.88)	2.19 (0.76)	0.21	2.48 (3.27)	2.21 (2.47)		0.61
MF	3.56 (0.82)	3.79 (0.86)	0.14	3.16 (0.75)	3.31 (0.75)	0.27	10.9 (7.77)	12.1(7.98)		0.36
ES	16.6 (2.76)	16.1 (2.59)	0.28	15.5 (2.59)	15.1 (2.42)	0.34	6.57 (4.39)	6.40 (3.58)		0.79
L3/4 level										
PM	9.20 (1.76)	9.12 (1.71)	0.80	9.05 (1.74)	8.98 (1.69)	0.82	1.45 (2.25)	1.36 (2.04)		0.81
QL	**3.69 (1.12)**	**3.30 (1.10)**	**0.047**	3.58 (1.12)	3.22 (1.07)	0.06	2.98 (4.26)	2.63 (3.12)		0.61
MF	5.81 (1.23)	5.96 (1.22)	0.48	4.93 (1.01)	5.05 (0.96)	0.50	14.8 (6.95)	14.9 (7.09)		0.88
ES	16.7 (2.70)	16.1 (2.56)	0.17	15.0 (2.63)	14.6 (2.43)	0.30	9.93 (5.53)	9.39 (5.09)		0.54
L4/5 level										
PM	12.4 (2.33)	12.1 (2.28)	0.53	12.2 (2.32)	12.0 (2.26)	0.58	1.17 (1.66)	0.90 (1.26)		0.30
MF	8.89 (1.49)	8.92 (1.44)	0.91	7.44 (1.37)	7.42 (1.21)	0.92	16.3 (6.93)	16.6 (6.82)		0.77
ES	15.3 (3.08)	14.7 (2.84)	0.26	13.4 (3.17)	12.9 (3.00)	0.38	12.4 (7.87)	12.3 (7.38)		0.91
L5/S1 level										
MF	11.2 (1.71)	11.1 (1.61)	0.65	8.99 (1.61)	8.95 (1.45)	0.89	20.0 (7.33)	19.2 (7.76)		0.50
ES	10.7 (3.46)	10.3 (2.81)	0.47	8.48 (3.20)	8.23 (2.67)	0.63	21.4 (11.5)	20.8 (10.6)		0.73
Males										
LI/2 level										
PM	4.27 (1.50)	3.84 (1.48)	0.13	4.21 (1.51)	3.79 (1.49)	0.14	1.39 (2.22)		1.52 (2.84)	0.79
QL	2.66 (1.00)	2.55 (0.94)	0.53	2.62 (0.99)	2.49 (0.93)	0.48	1.84 (2.90)		2.32 (2.69)	0.33
MF	2.72 (0.70)	2.84 (0.68)	0.38	2.60 (0.67)	2.71 (0.65)	0.37	4.28 (6.00)		4.35 (5.60)	0.94
ES	21.86 (3.32)	21.39 (3.38)	0.44	21.10 (3.18)	20.60 (3.29)	0.39	3.42 (2.47)		3.71 (3.01)	0.48
L2/3 level										
PM	9.61 (1.86)	9.76 (2.22)	0.70	9.54 (1.85)	9.67 (2.22)	0.72	0.71 (0.90)		0.92 (1.06)	0.25
QL	**4.53 (1.14)**	**4.03 (1.07)**	**0.02**	**4.46 (1.12)**	**3.96 (1.06)**	**0.02**	1.60 (1.39)		1.87 (1.67)	0.35
MF	4.38 (0.96)	4.58 (1.02)	0.28	4.09 (0.92)	4.26 (0.99)	0.34	6.53 (6.14)		6.88 (7.44)	0.77
ES	22.05 (3.40)	21.89 (3.64)	0.80	21.12 (3.33)	20.93 (3.52)	0.75	4.24 (2.36)		4.44 (2.57)	0.61
L3/4 level										
PM	14.65 (2.55)	14.45 (2.62)	0.67	14.54 (2.56)	14.35 (2.62)	0.68	0.72 (1.14)		0.65 (1.09)	0.73
QL	**6.35 (1.34)**	**5.83 (1.30)**	**0.04**	**6.25 (1.32)**	**5.74 (1.28)**	**0.04**	1.65 (1.38)		1.48 (1.74)	0.55
MF	6.98 (1.20)	7.41 (1.26)	0.07	6.42 (1.13)	6.78 (1.19)	0.10	7.70 (5.77)		8.47 (6.19)	0.51
ES	20.85 (3.57)	20.40 (3.81)	0.46	19.44 (3.52)	19.11 (3.67)	0.58	6.85 (4.19)		6.38 (4.18)	0.48
L4/5 level										
PM	18.83 (3.20)	18.10 (3.23)	0.22	18.70 (3.21)	17.99 (3.22)	0.23	0.75 (0.88)		0.53 (0.77)	0.14
MF	10.36 (1.69)	10.39 (1.75)	0.93	9.42 (1.65)	9.43 (1.71)	0.97	9.09 (5.84)		9.21 (6.26)	0.90
ES	18.01 (3.91)	17.71 (3.66)	0.66	16.26 (3.99)	16.25 (3.61)	0.99	10.02 (6.69)		8.42 (6.09)	0.13
L5/S1 level										
MF	12.62 (1.93)	12.54 (1.87)	0.83	10.87 (1.91)	10.93 (1.86)	0.86	13.94 (7.21)		12.88 (6.34)	0.34
ES	11.27 (5.04)	11.33 (4.57)	0.95	9.50 (4.51)	9.86 (4.30)	0.66	16.57 (9.36)		14.01 (8.19)	0.07

Adjusted for age and BMI.

Significant values are given in bold.

*CSA* cross-sectional area, *FCSA* functional cross-sectional area, *FI* fat infiltration, *PM* psoas major, *QL* quadratus lumborum, *MF* multifidus, *ES* erector spinae.

### Differences between left and right paraspinal muscles in the cLBP group

Table 3 reveals a similar asymmetry in muscle measurements between the left and right sides in both males and females in the cLBP group. In males, statistically significant differences were observed in the CSA of the QL at L2/3 and L3/4 (P < 0.05, Partial η^2^ > 0.04), as well as in the FCSA at L1/2 to L3/4 (P < 0.05, Partial η^2^ > 0.03). Additionally, compared to the no-BP group, the cLBP group exhibited a notable difference: both males and females demonstrated significantly higher FI values on the right side compared to the left in the ES at the L1/2 and L2/3 levels, with females also showing significant asymmetry at L3/4 (P < 0.05, Partial η^2^ > 0.03). No other statistically significant differences were identified.

**Table 3:** Differences between left and right paraspinal muscles in the cLBP group.

	CSA, mean (SD)		p value	FCSA, mean (SD)		p value	FI (%), mean (SD)		p value
	Left	Right		Left	Right		Left	Right	
Females									
LI/2 level									
PM	2.28 (0.88)	2.19 (0.96)	0.59	2.24 (0.86)	2.16 (0.96)	0.59	1.54 (1.80)	1.78 (2.26)	0.48
QL	1.76 (0.57)	1.65 (0.57)	0.24	1.73 (0.57)	1.60 (0.56)	0.18	2.19 (2.52)	3.12 (4.21)	0.11
MF	2.13 (0.44)	2.29 (0.50)	0.052	1.96 (0.43)	2.10 (0.48)	0.07	7.74 (8.63)	8.06 (8.27)	0.81
ES	16.23 (2.63)	15.84 (2.59)	0.31	15.31 (2.54)	14.72 (2.45)	0.10	**5.59 (4.04)**	**6.96 (4.32)**	**0.04**
L2/3 level									
PM	5.35 (1.17)	5.27 (1.28)	0.69	5.28 (1.14)	5.19 (1.26)	0.69	1.33 (1.40)	1.41 (1.53)	0.76
QL	2.70 (0.78)	2.48 (0.71)	0.08	2.64 (0.78)	2.41 (0.69)	0.053	2.20 (2.15)	2.84 (3.14)	0.15
MF	3.47 (0.81)	3.61 (0.84)	0.31	3.11 (0.71)	3.15 (0.73)	0.71	9.84 (8.95)	11.95 (9.93)	0.13
ES	17.09 (2.85)	16.87 (2.78)	0.60	15.85 (2.77)	15.37 (2.71)	0.23	**7.27 (4.57)**	**8.88 (5.25)**	**0.02**
L3/4 level									
PM	8.65 (1.55)	8.59 (1.57)	0.82	8.54 (1.54)	8.45 (1.55)	0.79	1.25 (1.29)	1.32 (1.27)	0.75
QL	3.89 (0.98)	3.61 (0.98)	0.07	3.79 (0.96)	3.51 (0.97)	0.06	2.53 (2.95)	2.55 (2.24)	0.96
MF	5.92 (1.14)	6.30 (1.30)	0.07	5.08 (1.01)	5.26 (1.11)	0.32	13.80 (8.27)	16.07 (8.88)	0.07
ES	16.94 (2.85)	16.45 (2.94)	0.27	15.03 (2.72)	14.32 (2.78)	0.09	**11.22 (5.81)**	**12.91 (6.40)**	**0.04**
L4/5 level									
PM	12.04 (1.86)	11.71 (1.76)	0.26	11.70 (1.84)	11.42 (1.76)	0.35	2.85 (2.62)	2.47 (2.41)	0.38
MF	9.17 (1.63)	9.42 (1.72)	0.36	7.20 (1.32)	7.32 (1.40)	0.90	20.87 (8.50)	22.78 (9.26)	0.14
ES	15.21 (2.96)	14.74 (2.74)	0.31	12.17 (2.83)	11.55 (2.72)	0.15	19.88 (9.28)	21.61(10.12)	0.19
L5/S1 level									
MF	11.58 (1.86)	11.81 (1.87)	0.47	8.61 (1.64)	8.60 (1.73)	0.97	25.52 (7.62)	27.00 (9.21)	0.24
ES	11.51 (3.66)	11.67 (3.95)	0.79	8.37 (3.42)	8.24 (3.81)	0.82	28.60 (11.67)	30.88 (12.04)	0.23
Males									
LI/2 level									
PM	4.58 (1.85)	4.35 (1.75)	0.45	4.49 (1.78)	4.27 (1.70)	0.44	1.67 (2.70)	1.74 (2.14)	0.87
QL	3.00 (0.93)	2.70 (0.94)	0.06	**2.94 (0.92)**	**2.62 (0.91)**	**0.04**	1.98 (2.09)	2.71 (2.48)	0.07
MF	2.84 (0.57)	2.96 (0.59)	0.25	2.73 (0.57)	2.84 (0.58)	0.26	3.84 (4.02)	3.83 (5.04)	0.98
ES	22.50 (3.13)	21.91 (3.16)	0.25	21.62 (3.07)	20.73 (3.16)	0.08	**3.94 (2.58)**	**5.42 (3.42)**	** < 0.01**
L2/3 level									
PM	10.04 (2.59)	9.98 (2.43)	0.89	9.90 (2.56)	9.86 (2.41)	0.93	1.46 (2.57)	1.17 (1.28)	0.40
QL	**4.73 (1.49)**	**4.19 (1.23)**	**0.02**	**4.65 (1.49)**	**4.11 (1.22)**	**0.02**	1.77 (1.85)	1.90 (2.09)	0.70
MF	4.58 (0.85)	4.76 (0.92)	0.27	4.32 (0.83)	4.45 (0.92)	0.38	5.63 (6.17)	6.43 (6.59)	0.40
ES	22.53 (3.12)	22.04 (3.02)	0.32	21.41 (3.18)	20.67 (3.21)	0.15	**4.99 (3.69)**	**6.36 (4.27)**	**0.02**
L3/4 level									
PM	15.23 (3.11)	15.14 (2.90)	0.67	15.15 (3.05)	15.00 (2.85)	0.76	1.20 (2.30)	0.91 (1.42)	0.38
QL	**6.17 (1.78)**	**5.59 (1.39)**	**0.03**	**6.04 (1.76)**	**5.50 (1.38)**	**0.04**	2.11 (3.61)	1.88 (2.47)	0.67
MF	7.40 (1.30)	7.61 (1.17)	0.34	6.77 (1.24)	6.88 (1.05)	0.60	8.37 (6.67)	9.25 (7.04)	0.42
ES	21.67 (3.47)	20.81 (3.31)	0.12	19.90 (3.55)	18.92 (3.29)	0.08	8.38 (5.70)	9.20 (5.62)	0.32
L4/5 level									
PM	19.66 (3.21)	19.35 (3.47)	0.55	19.12 (3.26)	18.88 (3.42)	0.65	2.76 (3.16)	2.37 (1.84)	0.36
MF	10.59 (1.69)	10.79 (1.52)	0.48	9.10 (1.51)	9.11 (1.40)	0.97	13.72 (8.18)	15.27 (8.50)	0.27
ES	17.56 (3.30)	16.85 (3.13)	0.19	14.75 (3.70)	14.07 (3.38)	0.23	16.61 (9.86)	17.05 (8.75)	0.75
L5/S1 level									
MF	13.21 (2.08)	13.31 (2.03)	0.78	10.68 (2.07)	10.73 (1.95)	0.89	19.23 (8.01)	19.44 (7.77)	0.87
ES	12.72 (4.54)	11.96 (3.63)	0.28	9.65 (4.46)	8.97 (3.60)	0.31	26.70 (14.09)	27.06 (12.70)	0.87

Adjusted for age and BMI.

Significant values are given in bold.

*CSA* cross-sectional area, *FCSA* functional cross-sectional area, *FI* fat infiltration, *PM* psoas major, *QL* quadratus lumborum, *MF* multifidus, *ES* erector spinae.

### Comparison of paraspinal muscle asymmetry indices between no-BP and cLBP groups

Compared to the female no-BP group, female subjects with cLBP had significantly higher CAI and FCAI of ES at the L5/S1 segment (p < 0.05), while the FCAI of PM at the L4/5 segment was lower (OR: 0.869, 95%CI: 1.008- 1.150, p = 0.03) (Table 4). Additionally, the female’s FIAI of ES at L2/3 and PM, MF, and ES at L4/5 was significantly higher in the cLBP group than in the no-BP group (p < 0.01).

**Table 4:** Comparison of paraspinal muscle asymmetry indices between no-BP and cLBP groups.

	CAI, OR (95%CI)	*p* value	FCAI, OR (95%CI)	*p* value	FIAI, OR (95%CI)	*p* value
Females						
LI/2 level						
PM	0.992 (0.941–1.045)	0.75	0.990 (0.939–1.042)	0.69	0.897 (0.713–1.130)	0.36
QL	1.043 (0.997–1.091)	0.07	1.041 (0.996–1.088)	0.08	1.081 (0.939–1.245)	0.28
MF	1.044 (0.967–1.127)	0.27	1.034 (0.948–1.128)	0.45	0.992 (0.927–1.062)	0.83
ES	0.929 (0.800–1.078)	0.33	0.987 (0.855–1.139)	0.86	1.176 (0.944–1.466)	0.15
L2/3 level						
PM	0.945 (0.874–1.023)	0.16	0.942 (0.870–1.020)	0.14	0.955 (0.607–1.502)	0.84
QL	1.034 (0.986–1.085)	0.17	1.036 (0.987–1.087)	0.16	1.078 (0.902–1.289)	0.41
MF	0.983 (0.902–1.070)	0.69	0.941 (0.842–1.052)	0.29	1.019 (0.939–1.106)	0.65
ES	1.067 (0.921–1.236)	0.39	1.120 (0.962–1.304)	0.15	**1.292 (1.020–1.637)**	**0.03**
L3/4 level						
PM	0.936 (0.846–1.037)	0.21	0.939 (0.847–1.041)	0.23	0.686 (0.390–1.205)	0.19
QL	0.976 (0.928–1.027)	0.35	0.981 (0.932–1.032)	0.46	0.807 (0.644–1.010)	0.06
MF	1.057 (0.959–1.165)	0.26	0.988 (0.890–1.098)	0.83	1.103 (0.968–1.258)	0.14
ES	0.893 (0.778–1.026)	0.11	1.027 (0.902–1.169)	0.68	1.128 (0.951–1.337)	0.17
L4/5 level						
PM	0.884 (0.782–1.000)	0.051	**0.869 (0.764–0.989)**	**0.03**	**1.619 (1.069–2.452)**	**0.02**
MF	1.081 (0.953–1.225)	0.23	1.035 (0.892–1.200)	0.65	**1.175 (1.017–1.356)**	**0.03**
ES	1.022 (0.913–1.143)	0.71	1.104 (0.987–1.235)	0.08	**1.221 (1.078–1.383)**	** < 0.01**
L5/S1 level						
MF	1.062 (0.943–1.195)	0.32	1.054 (0.887–1.253)	0.55	1.110 (0.987–1.248)	0.08
ES	**1.077 (1.008–1.150)**	**0.03**	**1.082 (1.004–1.165)**	**0.04**	1.076 (0.985–1.174)	0.10
Males						
LI/2 level						
PM	**0.939 (0.883–0.998)**	**0.04**	**0.937 (0.881–0.997)**	**0.04**	1.131 (0.848–1.508)	0.40
QL	1.108 (0.975–1.063)	0.42	1.019 (0.975–1.065)	0.41	0.935 (0.765–1.141)	0.51
MF	0.987 (0.900–1.082)	0.78	0.977 (0.881–1.084)	0.67	0.938 (0.809–1.087)	0.39
ES	1.069 (0.921–1.241)	0.38	1.095 (0.939–1.278)	0.25	**1.579 (1.155–2.158)**	** < 0.04**
L2/3 level						
PM	0.952 (0.876–1.034)	0.24	0.951 (0.875–1.034)	0.24	1.315 (0.852–2.031)	0.22
QL	1.014 (0.959–1.073)	0.62	1.018 (0.962–1.078)	0.53	1.085 (0.805–1.464)	0.59
MF	1.032 (0.940–1.133)	0.51	0.977 (0.880–1.086)	0.67	**0.849 (0.736–0.979)**	**0.02**
ES	1.045 (0.912–1.197)	0.53	1.057 (0.924–1.209)	0.42	**1.353 (1.017–1.801)**	**0.04**
L3/4 level						
PM	0.948 (0.842–1.068)	0.38	0.961 (0.853–1.082)	0.51	1.326 (0.786–2.237)	0.29
QL	1.039 (0.978–1.103)	0.21	1.038 (0.977–1.102)	0.23	0.801 (0.573–1.121)	0.20
MF	0.978 (0.882–1.084)	0.67	0.969 (0.864–1.086)	0.59	1.018 (0.876–1.183)	0.82
ES	1.060 (0.922–1.219)	0.42	1.067 (0.922–1.236)	0.38	0.907 (0.724–1.137)	0.40
L4/5 level						
PM	**0.864 (0.753–0.990)**	**0.04**	**0.849 (0.735–0.980)**	**0.03**	**2.925 (1.520–5.626)**	** < 0.01**
MF	1.084 (0.944–1.244)	0.25	1.028 (0.893–1.185)	0.70	1.054 (0.893–1.243)	0.53
ES	1.044 (0.947–1.150)	0.39	1.051 (0.947–1.167)	0.35	1.041 (0.943–1.149)	0.43
L5/S1 level						
MF	1.033 (0.907–1.178)	0.62	0.978 (0.848–1.128)	0.76	0.981 (0.847–1.135)	0.79
ES	0.971 (0.908–1.039)	0.39	0.974 (0.914–1.043)	0.47	1.015 (0.937–1.100)	0.71

Adjusted for age and BMI.

Significant values are given in bold.

*CAI* cross-sectional area asymmetry index, *FCAI* functional cross-sectional area asymmetry index, *FIAI* fat infiltration asymmetry index, *PM* psoas major, *QL* quadratus lumborum, *MF* multifidus, *ES* erector spinae, *OR* odds ratio, *CI* confidence interval.

In males with cLBP, the CAI and FCAI of PM at L1/2 and L4/5 were significantly lower compared to those in the group without back pain. Conversely, the FIAI of MF at L2/3 was lower than in the no-BP group (OR: 0.849, 95%CI: 0.736- 0.979, p = 0.02), while the FIAI of ES at L1/2 and L2/3 and PM at L4/5 was higher than in the no-BP group (p < 0.05).

### Relationship between paraspinal muscle asymmetry index and cLBP intensity and duration

In females, the FIAI of MF and ES at the L1/2 segment were positively correlated with the duration of cLBP pain (p < 0.05) (Table 5). The FIAI of PM at the L2/3 segment (beta = 0.434, 95%CI 0.004–0.864, Partial η^2^ = 0.06, p = 0.048) and MF at the L4/5 segment (beta = 0.130, 95%CI 0.016–0.243, Partial η^2^ = 0.07, p = 0.03) were positively correlated with the intensity of cLBP pain (Supplementary Table 2). No significant statistical differences were observed for the remaining segments (Supplementary Tables 3, 4, 5, 6).

**Table 5:** Relationship between FIAI and chronic low back pain duration.

	Beta coefficient (95% CI)	p	Partial η^2^
Females			
LI/2 level			
PM	1.172 (− 0.392–2.737)	0.14	0.03
QL	0.284 (− 0.319–0.887)	0.35	0.01
MF	**0.387 (0.021–0.753)**	**0.04**	**0.06**
ES	**1.285 (0.193–2.378)**	**0.02**	**0.08**
L2/3 level			
PM	0.289 (− 2.511–3.089)	0.84	< 0.01
QL	0.293 (− 0.639–1.225)	0.53	< 0.01
MF	0.245 (− 0.155–0.644)	0.23	0.02
ES	1.083 (− 0.021–2.187)	0.054	0.06
L3/4 level			
PM	0.469 (− 3.262–4.200)	0.80	< 0.01
QL	0.316 (− 0.978–1.611)	0.63	< 0.01
MF	0.204 (− 0.452–0.860)	0.54	< 0.01
ES	− 0.157 (− 1.104–0.790)	0.74	< 0.01
L4/5 level			
PM	− 1.675 (− 3.494–0.145)	0.07	0.05
MF	0.217 (− 0.959–0.525)	0.56	< 0.01
ES	0.052 (− 0.495–0.600)	0.85	< 0.01
L5/S1 level			
MF	− 0.024 (− 0.611–0.562)	0.93	< 0.01
ES	0.025 (− 0.440–0.490)	0.92	< 0.01
Males	Beta coefficient (95% CI)	p	Partial η^2^
LI/2 level			
PM	**2.046 (0.929–3.163)**	**0.001**	**0.18**
QL	− 0.627 (− 1.808–0.555)	0.29	0.02
MF	− 0.586 (− 1.293–0.122)	0.10	0.04
ES	0.889 (− 0.247–2.024)	0.12	0.04
L2/3 level			
PM	0.021 (− 1.163–1.205)	0.97	< 0.01
QL	0.057 (− 1.395–1.510)	0.94	< 0.01
MF	0.325 (− 0.487–1.136)	0.43	0.01
ES	1.099 (− 0.074–2.272)	0.07	0.06
L3/4 level			
PM	0.409 (− 1.119–1.938)	0.59	< 0.01
QL	− 0.214 (− 1.376–0.949)	0.71	< 0.01
MF	0.449 (− 0.309–1.206)	0.24	0.02
ES	0.152 (− 1.085–1.390)	0.81	< 0.01
L4/5 level			
PM	**1.203 (0.118–2.287)**	**0.03**	**0.08**
MF	− **0.743 (**− **1.419–-0.066)**	**0.03**	**0.07**
ES	0.193 (− 0.285–0.671)	0.42	0.01
L5/S1 level			
MF	− 0.174 (− 0.890–0.542)	0.63	< 0.01
ES	0.018 (− 0.358–0.394)	0.93	< 0.01

Adjusted for age and BMI.

Significant values are given in bold.

*FIAI* fat infiltration asymmetry index, *PM* psoas major, *QL* quadratus lumborum, *MF* multifidus, *ES* erector spinae, *CI* confidence interval.

In males, the FIAI of the PM at L1/2 and L4/5 was positively correlated with the duration of cLBP pain (p < 0.05). In contrast, the FIAI of the MF at L4/5 was negatively correlated with the duration of pain (beta = −0.743, 95%CI −1.419–−0.066, Partial η^2^ = 0.07, p = 0.03). Additionally, the CAI (beta = 0.100, 95%CI 0.032–0.168, Partial η^2^ = 0.13, p = 0.005) and FCAI (beta = 0.080, 95%CI 0.008–0.152, Partial η2 = 0.08, p = 0.03) of the ES at the L5/S1 segment were positively correlated with the intensity of pain (Supplementary Tables 7, 8, 9, 10, 11).

## Disscusion

This study highlights the presence of paraspinal muscle asymmetry between cLBP patients and no-BP individuals, with evidence indicating that these asymmetries differ between males and females. Specifically, significant asymmetry was observed in the CSA and FCSA of the QL in males and in the FI of the ES in cLBP patients. Furthermore, the CSA and FCSA of the left PM, QL, and ES were generally larger than their right-side counterparts, whereas the MF exhibited the opposite pattern. These findings are consistent with Niemelainen et al.^11^, who analyzed total CSA of paraspinal muscles in a normal population using MRI and found that the right MF is generally larger than the left, while the ES shows the opposite trend. This underscores the need for caution when interpreting asymmetry in paraspinal muscle size (CSA and FCSA) at specific levels and sides for identifying individuals with cLBP and spinal pathology.

However, in contrast to previous studies, our research reveals that cLBP patients exhibit more pronounced asymmetry in FI, particularly in the ES muscle, between the left and right sides of the paraspinal muscles compared to the no-BP group, across both males and females. Additionally, the significant difference in FIAI between the no-BP and cLBP groups suggests that asymmetric degeneration of paraspinal muscles is associated with cLBP. This underscores the potential role of asymmetric FI in the pathophysiology of cLBP. Higher FIAI values in the cLBP group indicate more severe muscle degeneration and asymmetry, potentially contributing to pain and functional impairment. These observations are consistent with previous research highlighting the role of muscle degeneration and asymmetry in the pathogenesis of cLBP^10,18^. Specifically, asymmetrical FI in cLBP patients predominantly affects the MF and ES muscles, with greater FI observed on the right side compared to the left. This asymmetry was not observed in the no-BP group, suggesting that greater FI in the right MF and ES muscles may contribute to the pathogenesis of cLBP.

Sex differences were evident in our study between individuals with cLBP and those without back pain. Both females and males in both groups exhibited left–right asymmetry, although the patterns varied across different muscles and lumbar segments. Furthermore, compared to asymmetry indices observed in the no-BP group, results differed significantly between females and males with cLBP. Specifically, females with cLBP showed significantly higher CAI and FCAI in the ES at L5/S1, indicating more pronounced asymmetry. In contrast, males with cLBP exhibited significantly lower CAI and FCAI in the PM at L1/2 and L4/5 levels compared to the no-BP group. These findings suggest distinct sex-specific patterns of muscle asymmetry, potentially influenced by factors such as muscle usage, hormonal influences, or other biological factors, which merit further investigation into their functional implications in clinical contexts.

Our study findings underscore a nuanced relationship between asymmetry indices of paraspinal muscles and cLBP. Across sexes, asymmetry indices in specific paraspinal muscles at distinct lumbar segments exhibited a positive correlation with the duration and intensity of cLBP. This implies that greater asymmetry between left and right sides in certain muscles at specific segments is associated with longer duration and higher intensity of cLBP. Notably, females tend to report higher pain intensity and significantly longer pain duration compared to males. This difference may stem from biological, psychological, and social factors influencing how males and females experience and report pain. Such differences could also contribute to the observed variations in the correlation between paraspinal muscle asymmetry indices and cLBP duration and intensity across sexes. While sex-specific analyses were performed to minimize the influence of these differences on the results, the limited sample size may have affected the study’s statistical power. Future research with larger sample sizes is needed to validate these findings and explore the underlying mechanisms in greater detail.

However, despite these correlations, most other asymmetry indices of paraspinal muscle did not show significant statistical relationships with the duration or intensity of cLBP. This may be attributed to Type I errors resulting from multiple tests. The absence of widespread and significant findings suggests that while muscle asymmetry may contribute to cLBP, it may not be the sole or primary determinant. Other factors, such as muscle strength^19,20^, endurance^21,22^, and neuromuscular control^23^, likely play critical roles and warrant further investigation. The hypothesis that patients with cLBP exhibit higher asymmetry indices of paraspinal muscles compared to no-BP individuals is supported by our findings. However, the assumption that paraspinal muscle asymmetry is directly related to cLBP remains uncertain.

These results align with existing literature, underscoring the complexity and multifactorial nature of cLBP^24,25^. Prior studies have identified various factors contributing to cLBP, including muscle composition and morphological changes, although muscle asymmetry has not been definitively established as a primary determinant. Future research should prioritize longitudinal studies to elucidate causal relationships and underlying mechanisms of these associations. Moreover, interventions into interventions aimed at mitigating muscle asymmetry could provide valuable insights into managing or alleviating cLBP.

However, several limitations inherent to our study warrant acknowledgement. Firstly, only 2.9% of cLBP patients experienced severe pain (VAS score ≥ 7), potentially limiting the generalizability of our findings to patients with severe cLBP. Furthermore, other relevant confounding factors, such as limb dominance, unilateral back pain, work activities, recreational and sports activities, and familial predispositions, were not systematically assessed in our study or may not have been adequately captured in existing data. These factors underscore the necessity for larger-scale studies to validate our findings comprehensively and address these potential limitations effectively.

## Conclusion

This study highlights the importance of sex-specific asymmetry in paraspinal muscles and its relevance for understanding cLBP. While muscle asymmetry appears relevant to cLBP, it is likely part of a multifactorial landscape that includes muscle strength, endurance, and neuromuscular control. Addressing these complexities through comprehensive treatment approaches tailored to mitigate muscle asymmetry could advance our understanding and management of cLBP. Future research should investigate the role of these asymmetries in contributing to cLBP and should develop comprehensive treatment approaches that integrate considerations of muscle asymmetry, strength, endurance, and neuromuscular control.

## Data Availability

The datasets generated during and/or analyzed during the current study are available.

## Abbreviations

cLBP: Chronic low back pain
no-BP: No back pain
MRI: Magnetic resonance imaging
PM: Psoas major
QL: Quadratus lumborum
MF: Multifidus
ES: Erector spinae
CSA: Cross-sectional area
FCSA: Functional cross-sectional area
FI: Fat infiltration
CAI: CSA asymmetry index
FCAI: FCSA asymmetry index
FIAI: FI asymmetry index
